# Modeling the Climatic Suitability of COVID-19 Cases in Brazil

**DOI:** 10.3390/tropicalmed8040198

**Published:** 2023-03-29

**Authors:** Jéssica Milena Moura Neves, Vinicius Silva Belo, Cristina Maria Souza Catita, Beatriz Fátima Alves de Oliveira, Marco Aurelio Pereira Horta

**Affiliations:** 1 Biosafety Level 3 Facility, Oswaldo Cruz Institute, Fiocruz, Rio de Janeiro 21040-900, Brazil; 2 Central-West Dona Lindu Campus, Federal University of São João del-Rei, Divinopolis 35501-296, Brazil; 3 Department of Geographic Engineering, Geophysics and Energy, University of Lisbon, 1749-016 Lisbon, Portugal; 4 Regional Office of Piauí, Fiocruz, Teresina 64001-350, Brazil

**Keywords:** coronavirus disease-19, climate, temperature, precipitation, humidity

## Abstract

Studies have shown that climate may affect the distribution of coronavirus disease (COVID-19) and its incidence and fatality rates. Here, we applied an ensemble niche modeling approach to project the climatic suitability of COVID-19 cases in Brazil. We estimated the cumulative incidence, mortality rate, and fatality rate of COVID-19 between 2020 and 2021. Seven statistical algorithms (MAXENT, MARS, RF, FDA, CTA, GAM, and GLM) were selected to model the climate suitability for COVID-19 cases from diverse climate data, including temperature, precipitation, and humidity. The annual temperature range and precipitation seasonality showed a relatively high contribution to the models, partially explaining the distribution of COVID-19 cases in Brazil based on the climatic suitability of the territory. We observed a high probability of climatic suitability for high incidence in the North and South regions and a high probability of mortality and fatality rates in the Midwest and Southeast regions. Despite the social, viral, and human aspects regulating COVID-19 cases and death distribution, we suggest that climate may play an important role as a co-factor in the spread of cases. In Brazil, there are regions with a high probability that climatic suitability will contribute to the high incidence and fatality rates of COVID-19 in 2020 and 2021.

## 1. Introduction

Coronavirus disease (COVID-19) has affected more than 250 million people worldwide [1]. In Brazil, the first imported case was confirmed on 25 February 2020 in São Paulo. After two years of the pandemic, Brazil topped fifth in the number of confirmed cases with 690,000 deaths until December 2022 [2]. A series of public containment policies, both at national and local levels, have been implemented since the beginning of the country’s pandemic. However, the pandemic has put pressure on the Brazilian health system, leaving public and private hospitals overwhelmed [3]. It was observed that emergency and intensive care units focused almost exclusively on patients with COVID-19, and in many cases, general and outpatient care was stopped to prevent the transmission of the virus [3,4].

The transmission of infectious diseases is determined by several factors, including social and economic factors, access to medical services, innate immunity, and ecological and climatic determinants [5]. Climate can influence the growth rate of pathogens [6] because their survival and reproduction rates depend on a favorable climate that facilitates their dissemination [7]. Previous studies have discussed the relationship between COVID-19 and environmental and climatic factors [8]. Most studies were based on climatic characteristics regarding exposure to the virus with the risk of pandemic propagation and concluded that the climate influences the spread of the pathogen [9,10]. The authors suggested that a cool, dry environment in a mesothermal climate is conducive to the spread of the coronavirus [11]. Recently, studies in Thailand, on the effects of meteorological factors have shown that the average temperature, relative humidity, wind speed, and absolute humidity have significant positive associations with the number of confirmed COVID-19 cases [12].

Environmental, climatic, and air pollution conditions have also been identified as possible co-factors for the lethality of SARS-CoV-2 [13]. The fatality rate in the Italian Lombardy region was 12%, while in the rest of Italy, it was 4.5% [14]. Researchers relate these differences to some of the main co-factors: the early implementation of social isolation measures, the availability of intensive care unit beds, under-reporting rates, different ways of notifying the number of deaths and infections between countries, and the proportion of older adults in the population [15]. However, this is an area of Europe with the highest air pollution because of its climatic conditions and geographic features, which cause the stagnation of pollutants [14].

Brazil is known to present complex meteorological scenarios, including equatorial, tropical, subtropical, temperate, semi-arid, and arid zones, which indirectly affect health problems related to the spread of several infectious respiratory diseases [4]. This is due to its extensive territory, population density, biomes, topography, and climate variability [8]. Given this and the need to understand the epidemiology of SARS-CoV-2, associated conditions, and exogenous variables, this study aimed to investigate the influence of regional climatic characteristics on the cumulative incidence of the period, mortality, and fatality of COVID-19 in Brazil, since there are no similar studies in the country that make this association over two years.

## 2. Materials and Methods

### 2.1. Health Data

**Figure 1:**
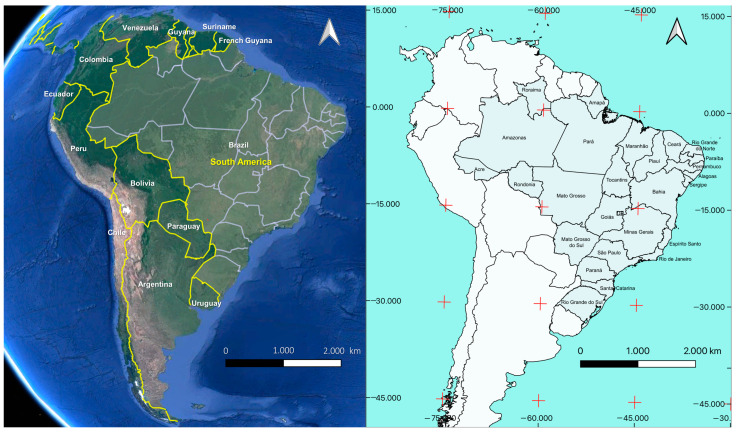
South America political map showing Brazil with international borders, neighboring countries and states.

We obtained notification records for COVID-19 cases and deaths for all Brazilian cities between 4 March 2020 and 31 December 2021 [16], as reported by State and Municipal Health Departments with corrections and updates in real time. The collected data were georeferenced to form pairs of central geographic coordinates (centroids) for each Brazilian municipality (Figure 1). All data were analyzed using a Microsoft Excel spreadsheet. For each Brazilian city, the cumulative incidence of COVID-19 (number of new cases per 100,000 inhabitants), mortality rate (MR) (frequency of death in the population per 100,000 inhabitants), and fatality rate (FR) (proportion of deaths among confirmed COVID-19 cases) was estimated for the study period. We distributed the variables in quartiles to transform the data into a binomial outcome because all algorithms used to model climate suitability require presence and absence to run as default settings for all modeling algorithms. Those municipalities that presented the 75% percentile for incidence, mortality rate, and fatality rate were selected as equivalent to high incidence (13,157.39/100,000 inhabitants), high mortality rate (294.27/100,000 inhabitants), and high fatality rate (2.9/100,000 inhabitants) and are described as 1 (one), indicating presence. Municipalities below the selected percentile are described as 0 (zero) in the database, indicating absence. All indicators were mapped.

### 2.2. Climate Data

Climate data including temperature, relative humidity, and precipitation were obtained from a digital database on the WorldClim website [17]. Information also included bioclimatic variables, which were derived from monthly values of temperature and precipitation and represented annual and monthly trends, seasonality, and extreme or limiting environmental factors (temperature of the coldest month or warmest month) that are important in species modeling analyses, ecological niches, and studies on climate change. The WorldClim variables include annual mean temperature (BIO1), mean diurnal range (BIO2), isothermality (BIO3), temperature seasonality (BIO4—standard-deviation 100), the maximum temperature of the warmest month (BIO5), minimum temperature of the coldest month (BIO6), annual temperature range (BIO7), mean temperature of the wettest quarter (BIO8), mean temperature of the driest quarter (BIO9), mean temperature of the warmest quarter (BIO10), mean temperature of the coldest quarter (BIO11), annual precipitation (BIO12), precipitation of the wettest month (BIO13), precipitation of the driest month (BIO14), precipitation seasonality (BIO15—coefficient of variation), precipitation of the wettest quarter (BIO16), precipitation of the driest quarter (BIO17), precipitation of the warmest quarter (BIO18), and precipitation of the coldest quarter (BIO19) [17]. We obtained georeferenced files with 19 bioclimatic variables corresponding to 1970–2000 in four spatial resolutions: 30 s to 2.5 min (0.93 × 0.93 = 0.86 km^2^ at the Equator), 5 min, and 10 min (18.6 × 18.6 = 344 km^2^ at the equator). The resolution selected for the model was 10 min (344 km^2^). Each download is a “zip” file containing 12 GeoTiff files, one for each month of the year (January to December), with image dimensions of (2160 × 1080 pixels) and 96 dpi resolution.

### 2.3. Data Analysis

To generate areas predicted to have a suitable environment for high COVID-19 incidence (HI), MR, and FR, an ensemble niche modeling approach based on seven different algorithms was implemented using the biomod2 package integrated into R software (R Foundation for Statistical Computing, Vietna, Austria, https://www.R-project.org/, accessed on 10 January 2022). Generalized linear regression model (GLM), generalized additive model (GAM), tree (CTA), flexible discriminant analysis (FDA), regression curve adaptive multivariate splines (MARS), random forest (RF), and maximum entropy (MAXENT) were used. The algorithms are based on the absence/presence datasets and estimate the environmental similarity between known places of species occurrence and regions that are still unknown. Thus, areas with greater similarity to known areas of occurrence were considered to have a high probability of occurrence.

For the modeling process, HI, MR, and FR were considered binomial outcomes (dependent variables), and bioclimatic variables were considered explanatory variables for adjusting the models. For each model run for the HI, MR, and FR outcomes, the bioclimatic variable with the highest percentage contribution to the seven predictive algorithms (GLM, GAM, CTA, FDA, MARS, RF, and MAXENT) was selected. We used the default settings for all algorithms with 10,000 pseudo-absences as background data for all algorithms. The models were created with training sets, and those with the best performance were selected. After selecting the bioclimatic variables that best fit the model performance, the area under the curve (AUC) evaluation metric of the receiver’s operational characteristics (ROC) was calculated AUC values range from 0 to 1, with 0.5–0.7 suggesting poor model performance, 0.7–0.9 indicating acceptable model performance, and >0.9 indicating excellent model performance. To generate the final ensemble models, we maintained models with ROC scores ≥ 0.8 [18].

We mapped the binary (0 and 1) distributions of the crude values of the dependent variables. After the modeling process using the seven algorithms, we obtained consensus maps with the ensemble models, where the climatic suitability was evaluated through an index ranging from 0 to 1, where 0 indicates regions with a low predicted probability of suitable climatic conditions (blue color) and 1 indicates regions with a high probability of suitable conditions (red color) for HI, MR, and FR. RStudio software version 1.4 was used with the packages biomod2 raster, rgdal, and ncdf4 [19]. Maps were generated using QGIS software version 3.16.

## 3. Results

**Figure A1:**
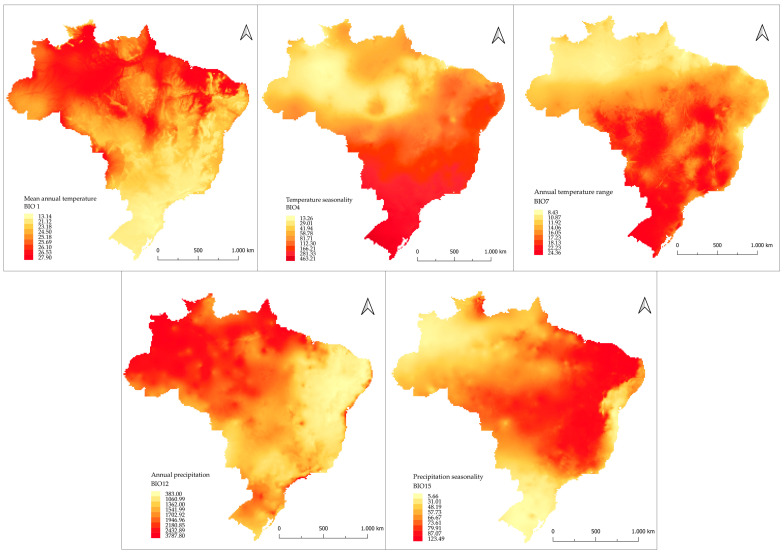
Distribution of climate variables used in modeling the climate suitability of COVID-19 cases in Brazil.

The contribution analysis of bioclimatic variables to HI indicated that the annual temperature range (BIO7), precipitation seasonality (BIO15), annual precipitation (BIO12), temperature seasonality (BIO4), and mean annual temperature (BIO1) were the most important variables shaping the incidence distribution in the models (all algorithms averaged) BIO7 made the highest relative contribution to this model (Figure A1). The contribution analysis of the MR indicated that the annual temperature range (BIO7), annual precipitation (BIO12), and temperature seasonality (BIO4) were the most important variables, with BIO7 showing the highest relative contribution (Table 1). Precipitation seasonality (BIO15), temperature seasonality (BIO4), and annual precipitation (BIO12) contributed the most to FR, with BIO15 exhibiting the highest relative contribution to the model.

**Figure 2:**
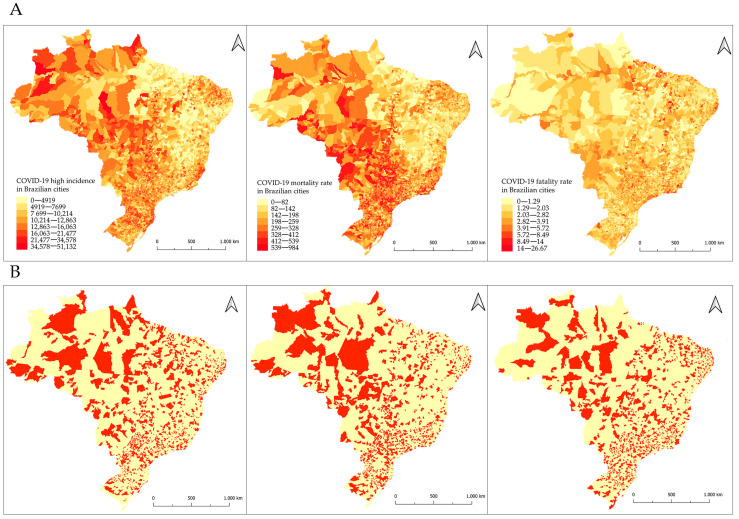
COVID-19 high incidence, mortality, and fatality rates in Brazilian cities (**A**). In (**B**), health indicators above the 75% percentile for COVID-19 in the years 2020 and 2021 in Brazil.

The map of COVID-19 HI, MR, and FR between 2020 and 2021 is shown in Figure 2A. The areas highlighted in red (cities) are those with values above the 75% percentile representing HI, MR, and FR. HI mapping showed an accumulation of COVID-19 notifications in the North, Midwest, South, and Southeast regions. The MR map highlights areas located in the North, Midwest, and Southeast regions. The FR map revealed that the country has areas with high fatality rates in different regions, mainly in the Midwest, Southeast, and Northeast (Figure 2B).

We observed a good fit for the models using the three outcomes (HI, MR, and FR). The average climate suitability consensus model for HI had an ROC value of 0.85 with a sensitivity of 84% and specificity of 72%. The mortality rate (MR) model had an ROC value = 0.88 for the modeling mean with a sensitivity of 76% and specificity of 83%. The FR model had an ROC = 0.82 for the modeling mean, with a sensitivity of 83% and specificity of 63% (Table 2).

**Figure 3:**
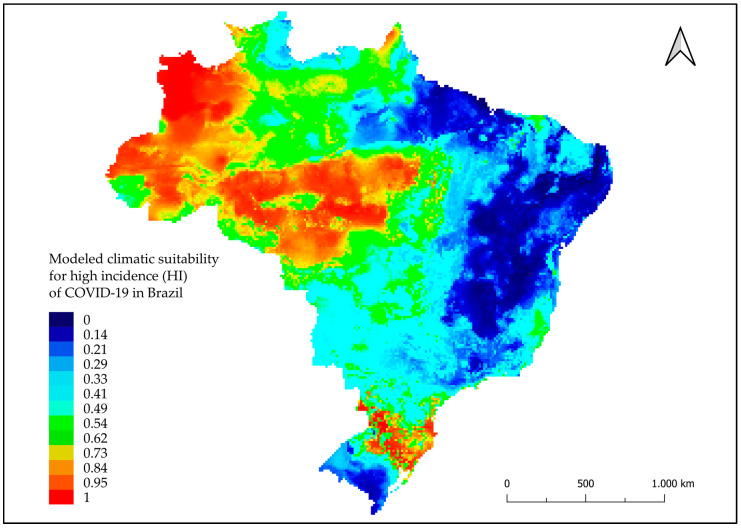
Modeled climatic suitability for high incidence (HI) of COVID-19 in Brazil. Modeled climatic suitability (consensus model) for all seven algorithms under current climate conditions. Data used for modeling was provided by WorldClim [17]. For visualization, maps were built using QGIS version 3.16 (https://qgis.org/pt_BR/site/, accessed on 10 January 2022).

**Figure 4:**
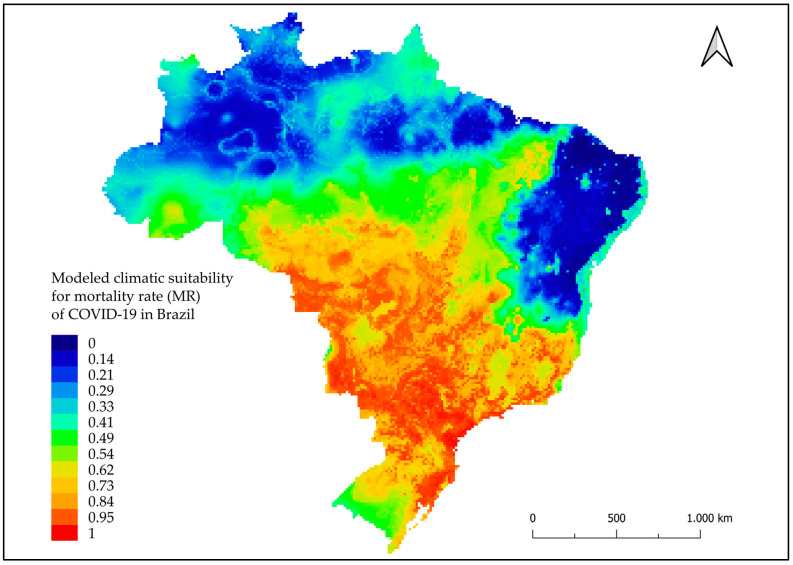
Modeled climatic suitability for mortality rate (MR) of COVID-19 in Brazil. Modeled climatic suitability (consensus model) for all seven algorithms under current climate conditions. Data used for modeling was provided by WorldClim [17]. For visualization, maps were built using QGIS version 3.16 (https://qgis.org/pt_BR/site/, accessed on January 10 2022).

**Figure 5:**
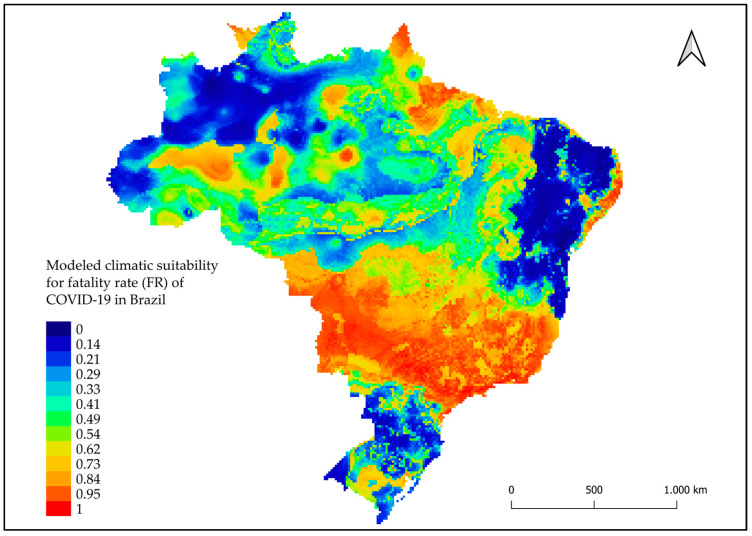
Modeled climatic suitability for fatality rate (FR) of COVID-19 in Brazil. Modeled climatic suitability (consensus model) for all seven algorithms under current climate conditions. Data used for modeling was provided by WorldClim [17]. For visualization, maps were built using QGIS version 3.16 (https://qgis.org/pt_BR/site/, accessed on 10 January 2022).

The HI climate suitability model showed areas highlighted in red on the map with points of high climate suitability (i.e., the greater likelihood that climate factors contribute to HI) mainly in the North and South regions and at some points in the Midwestern region (Figure 3). In the MR modeling, areas highlighted in red were observed, which were considered areas with high rates of climate suitability, mainly in cities in the Midwest, South, and Southeast regions (Figure 4). The FR model highlighted areas in cities in the Midwest, Southeast, and Northeast regions, and some in the North and South regions (Figure 5).

## 4. Discussion

Our results showed that predictive modeling for the three outcomes performed well, with an ROC curve > 0.80. The bioclimatic variable that performed best and influenced HI and MR analyses was the annual temperature range. This variable may contribute to the high incidence of COVID-19 in the North and South regions and the high mortality rate in the Midwest and Southeast regions. These regions have distinct climatic characteristics, suggesting that both warmer and colder areas may favor higher mortality outcomes from COVID-19, as they influence exposure, susceptibility, and the demands for emergency services [20]. The bioclimatic variable that achieved the best performance and influence in the FR model was precipitation seasonality, mainly covering the Midwest, Northeast, and Southeast regions. The seasonality of precipitation marks periods of drought and rain and is related to the regional temperature.

The economic development of some regions, mainly in the North and Midwest, with activities related to plant and mineral extraction and industrial and agro-industrial activities, has generated several environmental impacts, such as deforestation, fires, mining, logging, and artisanal gold mining, mainly in the Brazilian Amazon and Pantanal, which directly impact biome and its ecosystem services [21]. Such impacts have repercussions on climate change, human health, and the quality of life of affected populations, as they can influence the spread of several respiratory infectious diseases, such as SARS, influenza, and tuberculosis [22], which may explain the influence of the annual temperature range in the COVID-19 modeling of HI and MR.

A Brazilian study reported a cluster of cases in Manaus in the Amazon region in which the P.1 variant was identified in 42% of the specimens sequenced by the end of December 2020. In this region, approximately 75% of the population was infected with SARS-CoV-2 in October 2020. However, since mid-December, the number of cases has increased [23]. This mutation may be associated with the region’s impact on climate change and air pollution. In our study, we observed that predictive modeling for high incidence identified the Northern region of Brazil as the region with the highest probability that climatic factors, mainly annual changes in temperature, contributed to the increase in cases in that region. These changes not only directly affect the spatial distribution of zoonoses, introducing their hosts to new areas, but also lead to changes in species composition and ecology, which may result in new host–pathogen interactions that may create new transmission pathways or facilitate the evolution of harmful disease variants [24].

We highlight the South and Southeast regions when modeling climatic suitability. The economies of these regions are mainly based on industrial activities, mining, and agricultural production. The industrial sector is diversified, with greater expression in agro-industrial, metalworking, textiles, ceramics, machinery, and electronic equipment [25]. The South region is the leader in intensive pig and poultry farming in Brazil and has the largest herds in the country. The Southeast region has the most significant industrial sectors in the country, including automobiles, steel, petrochemicals, shipbuilding, and oil [26]. These activities cause major environmental impacts such as water, soil, and atmospheric pollution, deforestation of large areas, soil erosion, reduction of natural habitats, and extinction of fauna and flora species [27]. They produce an environmental transformation in the landscape that may directly impact climate and population health, which can cause diseases such as hepatitis, leptospirosis, and respiratory diseases such as flu and tuberculosis. They may contribute as an additional factor to the high incidence and mortality of COVID-19 [26].

Studies on climate suitability are necessary to analyze respiratory diseases because the effects of climate change can significantly impact people’s health status. Climate variability is an important factor in the spread of respiratory diseases as it influences the biological behavior of several disease-causing agents [28]. Changes in air pollution levels and climatic variables affect urban environmental health and often increase the likelihood of viral infection [29]. The key physical climate variables that are generally considered to affect the persistence of SARS-CoV-2 outdoors include air temperature, precipitation, relative humidity, wind speed intensity and direction, and solar radiation [30]. Air temperature is a leading environmental factor affecting seasonal and regional variations. The temperature has been shown to affect the duration of survival and transmission of SARS-CoV-2 through droplets, aerosols, and bioaerosols [31]. The modeling results of this study show that the annual temperature range is an important variable that contributes to the prediction that climate change may be an additional factor for the increase in the incidence of cases and mortality rate of COVID-19 in areas with different climates. Our study shows that despite presenting a complex meteorological scenario, temperature is an important co-factor for the spread of the virus in the environment. A study in China indicated a significant association between temperature and the daily incidence of COVID-19 based on local weighted regression and nonlinear models [32]. Research conducted in Madrid revealed that cold weather makes people more susceptible to daily viral infection transmission [33]. In Indonesia, a study of the population of Jakarta reported similar results, showing that the mean temperature was significantly correlated with the spread of COVID-19 [34]. A study conducted in seven metropolitan cities and nine provinces in South Korea also supported the link between COVID-19 incidence and temperature [35]. The studies described above demonstrate that temperature is an important co-factor in the spread of the virus, and our results reinforce this hypothesis.

In France, the emergence of the second COVID-19 wave coincided with a gradual drop in temperature despite the fall in social distancing and tended to reinforce the hypothesis of a significant effect of temperature on the incidence and death rate of COVID-19 [36]. Holtman et al. (2020) found that ambient temperature played a significant role in the spread of COVID-19 by promoting viral survival in low-temperature environments [9]. A study conducted in the temperate regions of the United States in 2022 demonstrated that above-average temperatures are consistently associated with a decrease in the relative risk (RR) of COVID-19 infection [37]. A Brazilian study found no correlations between daily maximum temperature and cumulative incidence or daily death rate of COVID-19 in Brazil [38], showing that the relationships between climate and COVID-19 and or other communicable diseases may not be so obvious as to be represented by simplistic linear models, something this study sought to avoid. Another Brazilian study showed that solar radiation, mean temperature, and wind speed were negatively correlated with the incidence of new cases [10]. Both studies analyzed climatic variables over a short period, which may have hindered the presence of a positive correlation with incidence and mortality. This does not corroborate the findings of this study, as the analyses were conducted considering the entire years of 2020 and 2021 and suggested a relationship between the selected climatic variables with cases and deaths of COVID-19. Other findings in Brazil have revealed that the coronavirus can adapt to Brazil’s subtropical and tropical climates [39]. Precipitation is positively associated with the incidence of COVID-19 [40]. Here, we observed the influence of seasonal precipitation on fatality rates in the Midwest and Southeast regions. These regions have a high rainfall index with two well-defined seasons (rainy summer and dry winter) and a semi-humid tropical climate. Seasonal cyclicity is an important feature of acute infectious diseases and is commonly observed in viral respiratory illnesses [41]. The authors reported that the same factors that drive the seasonality of flu-like illnesses cause the seasonality of COVID-19, such as temperature and relative humidity [42], corroborating the study. The predictive modeling analyses for the three outcomes included temperature seasonality and precipitation seasonality as explanatory variables, which performed well against bioclimatic variables. 

First, it was not possible to conduct more in-depth analyses of groups, age groups, and susceptible populations once we adopted a binary analysis for species niche modeling of COVID-19 cases, transforming data of presence and absence into high or low incidence, mortality, and fatality rates. Second, under-reporting at the beginning of the pandemic and other limiting factors of secondary databases, such as the reliability of records collected at a hierarchical level (city, state, country), may have somehow affected the findings of this study or under- or overestimated the probabilities estimated in this study. Third, the data are mapped based on mathematical models of climate variables and zones of likely climate suitability. However, other factors were not controlled, such as social vulnerability, the ability of the virus to mutate, the degree of social isolation, the proportion of elderly in the population, and the number of available hospital beds, among others. Further studies are needed on the effects of climate change on the dynamics of SARS-CoV-2, considering the various dimensions of the multi-causal universe of a pandemic. Although COVID-19 spread and its impact on population survival in recent years have been known to be influenced by social distancing, the presence of comorbidities, frequent viral mutations, use of masks, and PPE, factors such as geographical location, temperature, and precipitation may play an important role in disease transmission. Epidemiological evidence suggests that SARS-CoV-2 transmission risk is higher at lower ambient temperatures and lower humidity [43], and the climatic conditions observed in this study may affect virus survival, seasonal immunity, and population interaction.

## 5. Conclusions

Our study provides initial information on the influence of weather conditions on the transmission of COVID-19. Our results showed that the predictive modeling for the three outcomes performed well with an ROC curve > 0.80. The bioclimatic variable that performed best and influenced HI and MR analyses was the annual temperature range. This study reinforces the importance of more research involving climate factors, as temperature and rainfall regimes associated with pollution, UV radiation, and extreme weather events are significant factors affecting COVID-19 transmission in metropolises and must be included in future prediction models.

## Figures and Tables

**Table 1:** Values of the relative contribution of bioclimatic variables to modeling HI, MR, and FR by COVID-19 in 2020 and 2021, Brazil.

Dependent Variables	Bioclimatic Variables	Statistical Algorithms						
		GLM	GAM	CTA	FDA	MARS	RF	MAXENT
High incidence (HI)	Annual temperature range (BIO7)	0.647	0.519	0.326	0.323	0.519	0.361	0.550
	Precipitation seasonality (BIO15)	0.164	0.359	0.486	0.166	0.420	0.339	0.488
	Annual precipitation (BIO12)	0.206	0.303	0.436	0.141	0.390	0.272	0.448
	Temperature seasonality (BIO4)	0.010	0.177	0.524	0.629	0.087	0.280	0.045
	Mean annual temperature (BIO1)	0.081	0.118	0.510	0.128	0.120	0.202	0.097
Mortality Rate (MR)	Annual temperature range (BIO7)	0.709	0.320	0.359	0.283	0.286	0.499	0.333
	Annual precipitation (BIO12)	0.119	0.418	0.458	0.219	0.474	0.501	0.436
	Temperature seasonality (BIO4)	0.011	0.146	0.607	0.460	0.142	0.639	0.078
Fatality Rate (FR)	Precipitation seasonality (BIO15)	0.760	0.366	0.744	0.636	0.597	0.591	0.550
	Temperature seasonality (BIO4)	0.569	0.306	0.768	0.375	0.283	0.719	0.246
	Annual precipitation (BIO12)	0.478	0.286	0.541	0.274	0.372	0.532	0.380

**Table 2:** Evaluation of the modeling concerning the sensitivity and specificity of the ROC test for the period’s high incidence, mortality rate, and the fatality rate for COVID-19 in the years 2020 and 2021, Brazil.

Dependent Variables	Test ROC	Sensitivity	Specificity
High Incidence (HI)	0.851	84.321	72.167
Mortality Rate (MR)	0.881	76.723	83.490
Fatality Rate (FR)	0.821	83.067	63.098

## Data Availability

The data presented in this study are available on request from the corresponding author.
